# Large-Aperture kHz Operating Frequency Ti-alloy Based Optical Micro Scanning Mirror for LiDAR Application

**DOI:** 10.3390/mi8040120

**Published:** 2017-04-10

**Authors:** Liangchen Ye, Gaofei Zhang, Zheng You

**Affiliations:** State Key Laboratory of Precision Measurement Technology and Instruments, Department of Precision Instrument, Tsinghua University, Haidian District, Beijing 100084, China; ylc12@mails.tsinghua.edu.cn

**Keywords:** large-aperture, micro scanning mirror, micro scanner, Ti-alloy, LiDAR

## Abstract

A micro scanning mirror is an optical device used to scan laser beams which can be used for Light Detection and Ranging (LiDAR) in applications like unmanned driving or Unmanned Aerial Vehicle (UAV). The MEMS scanning mirror’s light-weight and low-power make it a useful device in LiDAR applications. However, the MEMS scanning mirror’s small aperture limits its application because it is too small to deflect faint receiving light. In this paper, we present a Ti-alloy-based electromagnetic micro scanning mirror with very large-aperture (12 mm) and rapid scanning frequency (1.24 kHz). The size of micro-scanner’s mirror plate reached 12 mm, which is much larger than familiar MEMS scanning mirror. The scanner is designed using MEMS design method and fabricated by electro-sparking manufacture method. As the experimental results show, the resonant frequency of the micro scanning mirror is 1240 Hz and the optical scanning angle can reach 26 degrees at resonance frequency when the actuation current is 250 mApp.

## 1. Introduction

LiDAR is widely used in many applications, such as space autonomous rendezvous docking, space target detection, UAV’s navigation, Advanced Driver Assistance System (ADAS), automatic driving and so on. These applications have recently created a great demand for low-cost, low-power and low-weighted three-dimension imaging LiDAR.

Micro-mirror based LiDAR have drawn the attention of many researchers for the realization of 3D distance measurement [1,2,3,4,5,6,7]. Traditional laser scanners for LiDAR consist of heavy, expensive and large rotational optical devices. In comparison with traditional laser scanning sensors, MEMS scanners have the advantages of rapid scanning frequency, light-weight and low power [8]. Micro-scanners’ advantages make it a promising technology for use in miniature LiDAR. However, current MEMS scanning mirrors’ mirror plates are too small to be applied in LiDAR applications in order to detect receiving light. In LiDAR applications, large apertures are required for the measuring beam [9]. The range of the LiDAR is largely affected by the size of the mirror which reflects the received laser light. The size of mirror plate must be big enough to gather more light scattered by the target. Most MEMS scanners are designed in nearby applications like projectors or Optical coherent tomography (OCT) and cannot meet the requirements of LiDAR applications.

Efforts have been made to achieve large-aperture MEMS scanning mirrors. Sandner et al. [5] present a 1D-MEMS scanner module which has a resonant frequency of 250 Hz and a mirror plate size of 2.52 × 9.51 mm^2^ per single mirror element. Lei et al. [10] present an electrothermal MEMS scanning mirror with a large aperture of 10 × 10 mm^2^ which has a resonant frequency of 234 Hz. Milanovic et al. [11,12] present a gimbal-less Tip–Tilt–Piston MEMS scanner with a mirror plate size of 5 mm and a resonant frequency of 334 Hz.

However, current large-aperture MEMS scanner frequency is not rapid enough to achieve fast LiDAR images and the aperture is not large enough. Silicon is a material with a very high strength but its fatigue strength is much lower than its yield strength [13]. The fatigue strength decreases when the size of MEMS structure increases [14]. The metal based micro scanner is more robust than silicon-based MEMS devices due to its ductile properties in comparison with brittle silicon substrates [15]. Some metallic materials like stainless-steels have been researched to be able to replace the silicon substrate of a MEMS scanner due to the ductile properties in comparison with the brittle Si materials. Park et al. [15] presents a one-axis metal-based micro-scanner with a large mirror (3 × 3 mm^2^), with a resonant frequency of 304 Hz and a scanning angle of 12 degrees. Youmin et al. [16] present a two-axis soft-magnetic stainless steel based micro scanner with a large-aperture of 4 × 5 mm^2^ which has frequency of 112 and 1268 Hz in each axis.

In this paper, we present a novel one-axis Ti alloy-based electromagnetic micro scanning mirror with a very large-aperture and a high operating frequency. Ti-alloy substrate is used to achieve large aperture and fast scanning frequency. The size of the micro scanning mirror can reach 12 mm with kHz resonant frequency. The mirror substrate is fabricated by electro-sparking manufacture technology. A moving coil is attached to the back of the mirror and a pair of moon-like magnets are employed to achieve a large optical scanning angle a low actuation current. A Position Sensitive Device (PSD) is integrated to measure the rotation angle of the mirror plate. The design, simulation, fabrication and characterization of the micro-scanner is described in this article.

## 2. Micro Mirror Based LiDAR System

**Figure 1:**
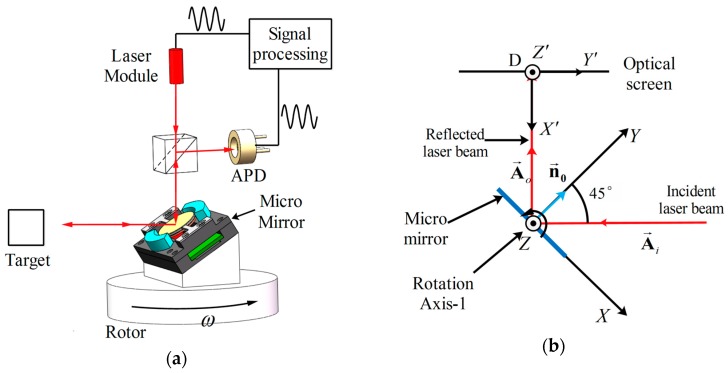

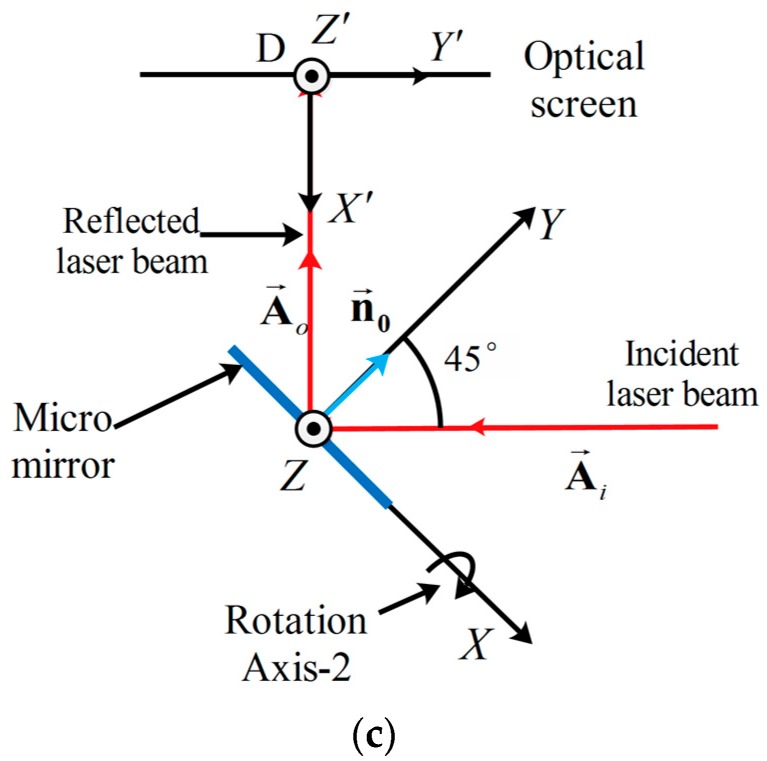
(**a**) Setup of two-axis Light Detection and Ranging (LiDAR) with 1D micro scanning mirror; (**b**) 1st arrangement of LiDAR system; (**c**) 2nd arrangement of LiDAR system.

Figure 1a shows a concept for a one-axis micro scanning mirror based LiDAR. One-axis scan is realized by the motor’s 360° rotation. The micro mirror scans the laser beam in another axis. The distance between the micro mirror and the target is determined by measuring the phase delay between the laser emitted and the laser received. To gather more reflected laser signal from the target, a larger-aperture micro mirror is required. To improve the horizontal resolution of the LiDAR image, the micro mirror needs to achieve fast operating frequency. Traditional scanners, like high-speed Galvano mirrors, are not suitable for this system because of their large size and slow, heavy weight. A kHz resonant frequency micro mirror with 12 mm aperture is required in this LiDAR system.

Figure 1b,c show the normal arrangement of the LiDAR system. The rotational axis of the micro mirror can be set along the *Z*-axis (called the 1st arrangement) or along the *X*-axis (called the 2nd arrangement). The 1st arrangement is chosen in our LiDAR system for the curve-shaped field distortion caused by the 2nd arrangement.

The intersection point $\left(x_{D},y_{D},z_{D}\right)$ of the reflected laser beam and the screen can be calculated by solving the below equation:

(1) $$\{\begin{array}{c} (x_{D},y_{D},z_{D})=a{\vec{Α}}_{o}\\ y_{D}-x_{D}=\sqrt{2}l \end{array}$$

where *l* is the distance between the micro mirror and the optical screen and ${\vec{A}}_{o}$ is the vector the of reflected laser beam, which can be calculated by solving the equation of reflection:

(2) $${\vec{A}}_{o}={\vec{A}}_{i}-2{\vec{n}}_{0}({\vec{n}}_{0}\cdot {\vec{A}}_{i})$$

where ${\vec{A}}_{i}$ is the vector of the incident laser beam and ${\vec{n}}_{0}$ is the normal vector of the mirror plate.

The intersection point’s coordinate $\left(x_{D1}^{\prime },y_{D1}^{\prime },z_{D1}^{\prime }\right)$ in the 1st arrangement and $\left(x_{D2}^{\prime },y_{D2}^{\prime },z_{D2}^{\prime }\right)$ in the 2nd arrangement can be calculated by coordinate transformation as:

(3) $$\{\begin{array}{l} x_{D1}^{\prime }=z_{D1}^{\prime }=0\\ y_{D1}^{\prime }=-l\tan2\alpha \end{array}$$

(4) $$\{\begin{array}{l} x_{D2}^{\prime }=0\\ y_{D2}^{\prime }=l\tan^{2}\beta \\ z_{D2}^{\prime }=\sqrt{2}l\tan\beta \end{array}$$

where α and β are scanning angles of the mirror plate at the 1st arrangement and the 2nd arrangement separately.

From Equation (3), we can see that the laser beam length ($y_{D1}^{\prime }$) in the 1st arrangement is about $\sqrt{2}$ times larger than the length ($z_{D2}^{\prime }$) in the 2nd arrangement. In addition, the laser beam in the second arrangement is curve-shaped for the y-coordinate of the intersection $y_{D2}^{\prime }\neq 0$. In our LiDAR, the 1st arrangement is occupied.

## 3. Design and FEM Simulation of the Micro Mirror

**Figure 2:**
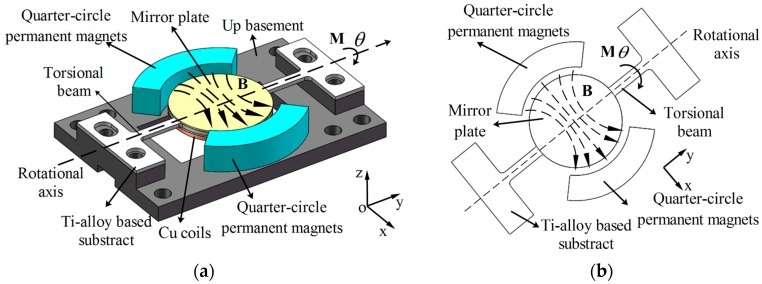
(**a**) Sketch of electromagnetic Ti-alloy based micro-scanner; (**b**) Principle of the electromagnetic micro-scanner.

**Figure 3:**
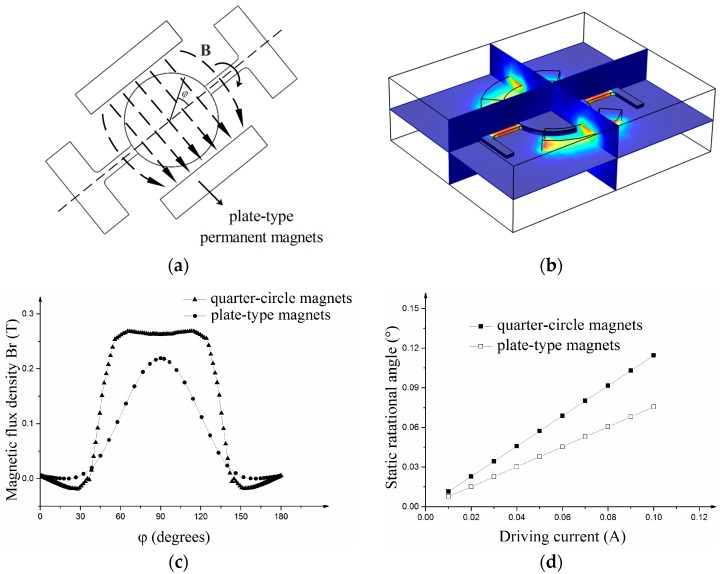
(**a**) Sketch of electromagnetic micro scanning mirror with plate-type magnets; (**b**) magnetic field-structure coupling Finite Element Method (FEM) simulation model of micro mirror; (**c**) magnetic flux density along direction of coil’s radius ($B_{r}\left(\varphi \right)$) in micro mirror with quarter-circle magnets in comparison with one with plate-type magnets; (**d**) relationship between static rotational angle and actuation current of micro mirror with quarter-circle magnets against one with plate-shape magnets.

Figure 2a shows a sketch of the electromagnetic Ti-alloy based micro scanning mirror. The scanner consists of an Ag-coated mirror plate, Ti-alloy based mirror substrate, moving coils, permanent magnets and Al-alloy basement. The micro mirror is actuated by electromagnetic torque. Figure 2b shows the principle of the electromagnetic micro-scanner. The scanner is actuated by the magnetic torque along the torsional beam. The torque is generated by the interaction between the permanent magnets and the AC-excited coils on the back of the mirror plate. A pair of quarter-circle magnets (see Figure 2a) is applied to enhance the scanning angle of micro-scanner. Compared with flat magnets (see Figure 3a), quarter-circle magnets can achieve larger magnetic torque (see below discussion).

When operating, the micro-scanner rotates along the rotational axis and the torsional angle (*θ*) is the only Degree of Freedom (DOF) in this dynamic system. The equation of the motion of the 1-DOF can be estimated as:

(5) $$I\ddot{\theta }+D\dot{\theta }+K\theta =M$$

where *I* is the moment of inertia of the scanning mirror, *D* is the damping coefficient, *K* is the stiffness of the torsional beam and *M* is the torque generated by the coil’s interaction with off-chip magnets.

The mirror of the micro-scanner contains a Ti-alloy substrate, a SiO_2_ based mirror plate and a Cu based multi-turns coil. The moment of inertia can be written as:

(6) $$I=\frac{1}{4}\rho_{s}\pi R_{s}^{4}t_{s}+\frac{1}{4}\rho_{m}\pi R_{m}^{4}t_{m}+\frac{1}{4}\rho_{c}\pi (R_{c1}^{4}-R_{c2}^{4})t_{c}$$

where $\rho_{s}$, $R_{s}$ and $t_{s}$ are the density, radius and thickness of mirror substrate, $\rho_{m}$, $R_{m}$ and $t_{m}$ are the density, radius and thickness of mirror plate, and $\rho_{c}$, $R_{c1}$, $R_{c2}$ and $t_{c}$ are the density, internal radius, external radius and thickness of the coil.

Followed by the formula reported in [17], the spring constant *K* is given as:

(7) $$K=\frac{G_{yx}wh^{3}}{3l}(1-\frac{192}{\pi^{5}}\frac{h}{\mu w}\tanh\frac{\pi w}{2h}),\mu =\sqrt{G_{yx}/G_{yz}}$$

where *w*, *h* and *l* are width, depth and length of torsional beam and $G_{yx}$ and $G_{yz}$ are shear moduli in different direction due to material anisotropy.

When operating, the micro-scanner is working on its resonant frequency which can be given as:

(8) $$f=\frac{\sqrt{K/I}}{2\pi }$$

According to the parameters in Table 1, the resonance frequency of the micro scanner is 1141 Hz.

The amplitude of the scanning angle at resonant frequency can be estimated as:

(9) $$\theta (\omega_{n})=\frac{M}{2\xi I}$$

where ξ is damping ratio which can be calculated as $\xi =D/2\sqrt{KI}$. Damping ratio is 0.0011 which can be calculated by the drag air damping model [18].

According to Equation (9), the amplitude of the scanning angle can be obviously enhanced by enlarging the torque generated by the magnetic field. The force acting on a current conductor in the magnetic field can be given as:

(10) dF=Idl×B

where *I* is the current running through the path (dl) and **B** is the external magnetic field.

Since the coil is a round loop, the torque (**M**) generated by the coil’s interaction with off-chip magnets can be estimated by integrating force along the coil:

(11) $$M=N\oint_{L}r\times (Idl\times B)$$

where *N* is the number of coil turns and **r** is the vector from location of dl to the rotational axis.

When the permanent magnets are plate-type magnets, the magnetic field between the permanent magnets is constant (see Figure 2a). Then Equation (6) can be simplified as:

(12) $$M=N\int_{0}^{2\pi }BIrd\theta \sin(\theta +\frac{\pi }{2})r\cos\theta =NBI\pi r^{2}$$

where *I* is coil current, *B* is the external magnetic field, and *r* is radius of the coil, respectively.

To increase the torque generated by the magnetic field, quarter-circle magnets are applied in the micro-scanner. The distance between the coil and the magnets can be drawn closer and more torque can be gained on the coils. Accounting for the symmetry of the coil, Equation (11) can be simplified as:

(13) $$M_{y}=N\oint_{L}r_{c}\sin\varphi I_{c}B_{r}(\varphi )$$

where $M_{y}$ is the torque along the direction of torsional beam, $I_{c}$ is the actuation current and $B_{r}$ is component of magnetic flux density along the direction of the coil’s radius.

A magnetic field-structure coupling Finite Element Method (FEM) simulation is conducted to analyze the interaction between the magnets and the micro-scanner with a coil (see Figure 3b). In the FEM simulation, the height, thickness and length of the two magnets is the same and the minimum distance between magnets and the mirror is the same. As the result of magnetic simulation show, the component of magnetic flux density along the direction of the coil’s radius ($B_{r}\left(\varphi \right)$) in the micro-scanner with quarter-circle magnets is larger than that in the micro-scanner with plate-type magnets (see Figure 3c). In the magnetic field-structure coupling simulation, the same actuation current is applied to analyze the static rotational angle according to magnetic force. The micro-scanner with quarter-circle magnets can achieve a larger rotational angle than the scanner with plate-type magnets (see Figure 3d). More magnetic force can be generated when applying the quarter-circle magnets.

**Figure 4:**
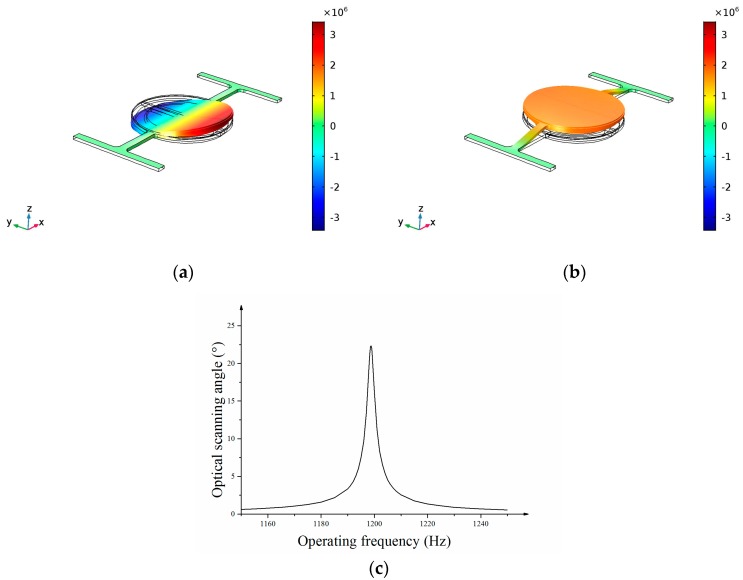
Modal analysis results from FEM simulation (**a**) Torsion mode of the micro mirror (scan mode); (**b**) piston mode of the micro mirror; (**c**) frequency response of the optical scanning angle in the twisting mode.

Figure 4 shows the modal FEM simulation results of the 1-D micro scanning mirror. The torsional resonant mode and piston resonant mode are shown in Figure 3a,b. The frequency of torsional mode is 1199 Hz and the frequency of piston mode is1917 Hz. The torsional mode is selected as the operating mode. The frequency of the piston mode is designed much higher than that of torsional mode to avoid interference with scan mode. Figure 4c shows the frequency response of the optical scanning angle in the twisting mode. The optical scanning angle at the resonance frequency is 22.4° at an actuation current of 140 mApp.

## 4. Packaging and Integration of Micro Scanning Mirror

**Figure 5:**
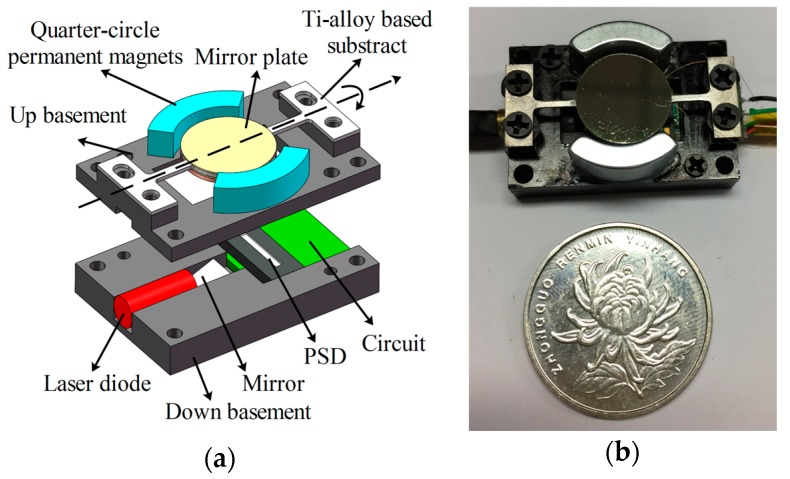
(**a**) Sketch of micro scanning mirror’s structure; (**b**) package of the micro scanning mirror.

Figure 5a shows the sketch of the electromagnetic Ti-alloy based micro scanning mirror module. The module consists of the Ag-coated mirror plate, the Ti-alloy based micro-mirror substrate, the cooper coils and the NdFeB-type permanent magnets (R11*90°, Jinchen Co. Ltd., Shenzhen, China), Position Sensitive Device (PSD, BS-PSD0018, Bosen Tech., Wuhan, China) and Al-alloy basement. The Ti-alloy based micro-mirror substrate is fabricated by electro-sparking manufacture method and the Al-alloy basement is fabricated by numerical control processing technology. The Ag-coated mirror plate is fabricated by spurting Ag on SiO_2_ substrate. Cooper coils are winded and the diameter of cooper wires is 50 μm. Ag-coated mirror plate and cooper coils are fixed on the Ti-alloy substrate using 3M instant adhesive glue (CA40H Minnesota Mining and Manufacturing Company, St. Paul, MN, USA). The micro scanning mirror is actuated by electromagnetic torque. The electromagnetic actuation consists of a pair of permanent magnets which are fixed on the basement using CA40H glue and a moving coil attached to the back of the mirror substrate. When applying a sine waveform current, the coil yields a torque along the torsional beam and actuates the mirror plate rotating along the beam. Beneath the mirror plate, a PSD device fixed in the substrate is used to measure the rotational angle of the mirror plate by sensing the position of the laser point on PSD reflected by the mirror on the back of the mirror plate. When the mirror plate rotates at different angles, the laser point reflected by the mirror on the back will fix at a different position on the PSD which will lead to an output voltage linear to the position. Figure 5b shows the package of the micro scanning mirror. The whole chip size is 31.6 × 21 × 8.5 mm^3^ and the weight is 18 g.

## 5. Experimental Results

In this section, features of the micro scanning mirror were measured. Operating frequency, optical scanning angle and angle measurement precision were the key characters of the micro scanning mirror. One-axis detection with the micro mirror was also achieved.

**Figure 6:**
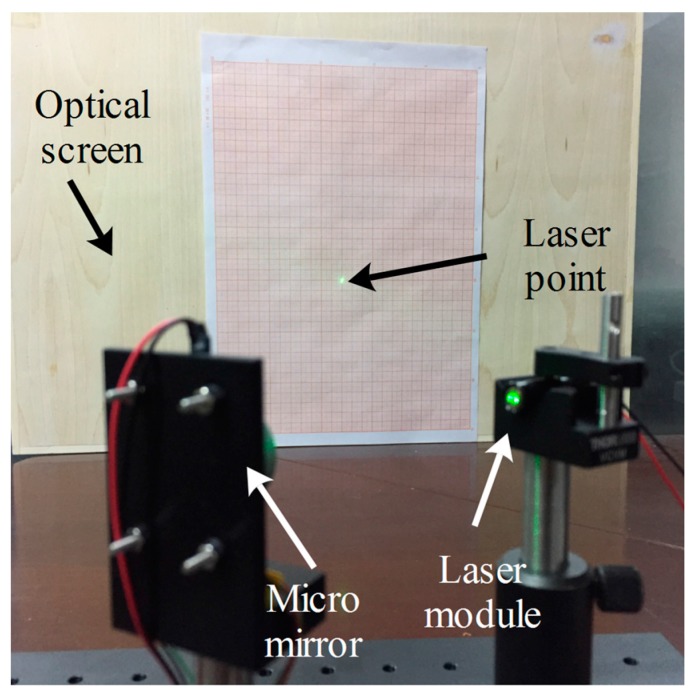
Experimental setup of micro mirror’s character measurement.

Figure 6 shows the experimental setup to measure the micro mirror’s features. The laser beam reflected by the micro mirror radiates toward the optical screen and results in a laser line on the screen. The optical scanning angle of the micro mirror can be achieved by measuring the length of the laser line and the distance between the micro mirror and optical screen (see Equation (3)).

**Figure 7:**
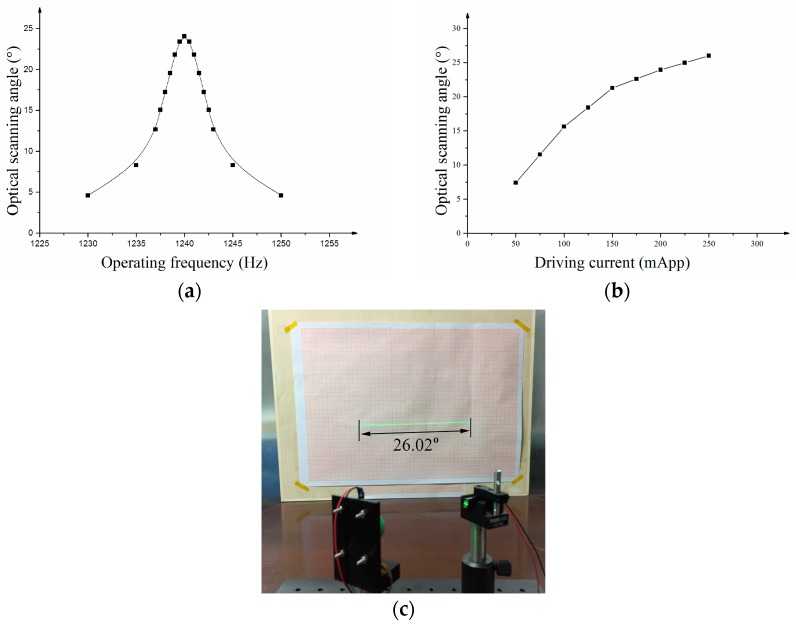
(**a**) Curve of optical scanning angle-versus-frequency; (**b**) curve of optical scanning angle versus actuation current; (**c**) maximum optical scanning angle of micro mirror.

Figure 7a illustrates the relationship between optical scanning angles with the operating frequency when the actuation current is 200 mA. The resonant frequency of the micro mirror was 1.24 kHz and the quality factor *Q* was 253. Figure 7b illustrates the relationship between the optical scanning angles with the actuation current of the coils. When the actuation current was 250 mApp, the micro mirror achieved the maximum scanning angle of 26 degrees (see Figure 7c). 

**Figure 8:**
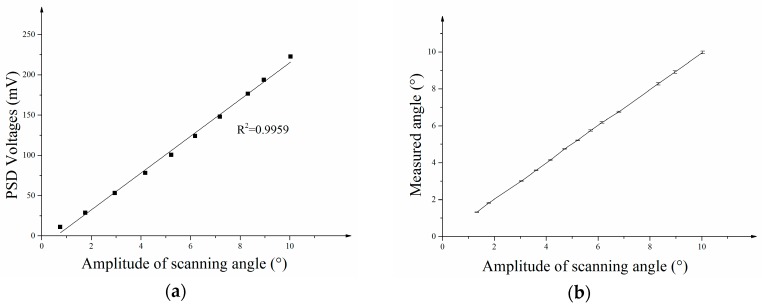

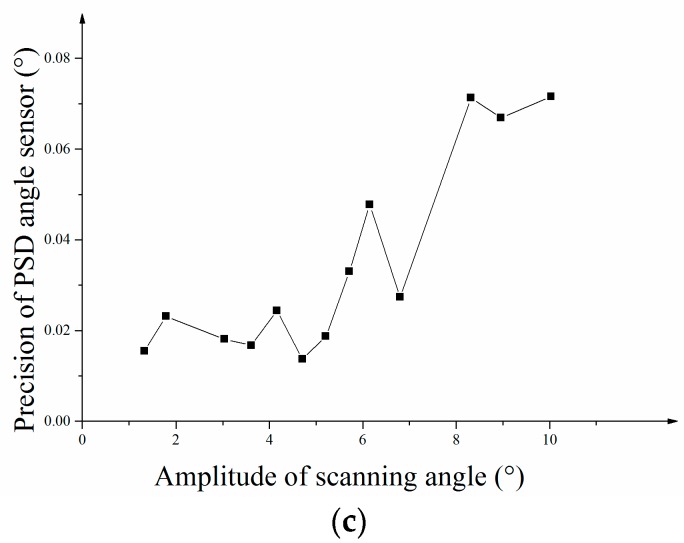
(**a**) The relationship between PSD Voltage and Optical scanning angle; (**b**) the relationship between measured angle and amplitude of scanning angle; (**c**) 3*σ* errors at each scanning angle.

The optical scanning angles were measured by the PSD optical device integrated in the package (see Figure 5a). An I-V conversion chip was applied to transform PSD’s current signal into a voltage signal. Figure 8a shows the relationship between the PSD Voltages and the optical scanning angle. The relationship is linear and the correlation coefficient *R*^2^ is 0.9959. The maximum amplitude of the scanning angle measurement in the experiment is 10 degrees while the whole optical scanning angle is 20 degrees. Amplitude of the scanning angle was measured by the PSD sensor and the precision was measured. Figure 8b shows the relationship between the measured angle and the amplitude of scanning angle with y-errors on the curve. Each scanning angle was measured nine times. The precisions of angle measurements at different scanning angles were calculated by achieving the 3*σ* error (thrice standard error) of each angle. Figure 8c shows the 3*σ* errors at each scanning angle and the maximum 3*σ* error (thrice standard error) of angle measurement is 0.07° at 10 degrees.

**Figure 9:**
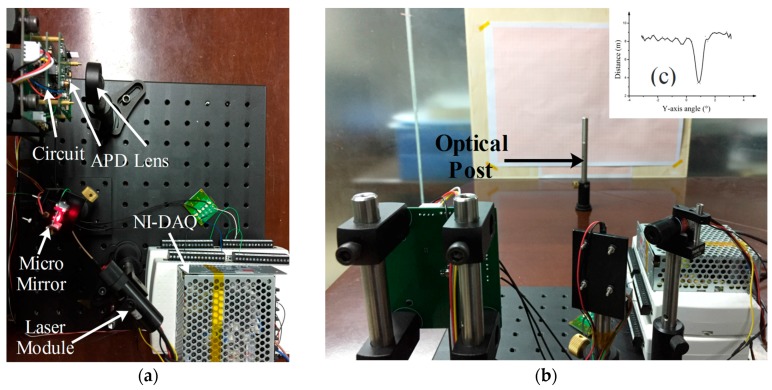
(**a**) Experimental setup of one-axis detection with micro mirror; (**b**) arrangement of LiDAR system and target; (**c**) one-axis detection picture of an optical post.

The micro mirror was occupied in a one-axis LiDAR and one-axis detection was achieved. Figure 9a shows the setup of the LiDAR system. Laser was deflected by the micro mirror to the target and the angle of the target was measured by the mirror. The distance of the target was measured by the laser rangefinder module. The rangefinder module could detect the distance by measuring the phase delay between the output laser beam and the reflected laser beam. Figure 9c shows the one-axis detection picture of an optical post.

**Figure 10:**
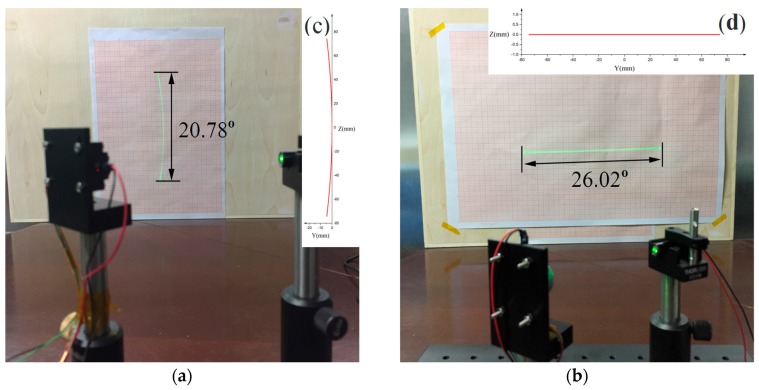
(**a**) Curve-shaped field distortion caused by arrangement 1 of micro mirrors; (**b**) scanned laser line on the screen in the 2nd arrangement; (**c**) simulation of curve-shaped field distortion caused by arrangement 1; (**d**) simulation of scanned laser line on the screen in the 2nd arrangement.

From the experimental results, we can see that in the first arrangement (see Figure 10a) the laser line scanned by the micro mirror is curve-shape distorted which will make our imaging solution complex (see Equation (4)). However, in the 2nd arrangement (see Figure 10b), the laser line is straight so that the imaging solution is simple (see Equation (3)). The experimental results fit our simulation results well.

## 6. Discussion

It’s very difficult to design a large-aperture and fast operating frequency micro mirror. From Equations (6)–(8), the resonance frequency can be calculated as:

(14) $$f=k_{size,1}\sqrt{\frac{G}{\rho }}$$

where *k_size_*_,1_ is a coefficient which is only related to the size of the structure. The ratio of shear modulus and density ($\sqrt{G/\rho }$) of the material itself determines the mechanical resonance frequency. It is similar to the effect of the ratio of Young's modulus and density ($\sqrt{E/\rho }$) [15], because Young modulus (*E*) and shear modulus (*G*) has the relationship of *G* = *E*/(2 + 2*ν*) and poison ratios of most common materials are between 0.2 and 0.4.

On the other hand, the maximum shear stress of the micro scanner should be maintained to be less than the strength of the material. The maximum shear stress of the micro-scanner can be given as [19]:

(15) $$\tau_{\max}=\frac{\beta Gw}{2\alpha l}\theta_{\max}\leq [\tau ]$$

where *α* and *β* are coefficients related to the width (*w*) and depth (*h*) of the beam [20] and [*τ*] is the shear strength of material.

When designing a larger micro-scanner, the moment of inertia becomes larger and the resonance frequency decreases. From Equation (7), to increase the resonance frequency of the large-aperture micro-scanner, the width (*w*) and the depth (*h*) should be increased and the length *l* should be decreased. From Equation (14) we can see that the shear stress will be increased which will lead to a decrease in maximum scanning angle. It is difficult to design a micro-scanner with large-aperture and large resonance frequency at the same time.

Table 1 lists the parameters of some common materials. The ratio $\sqrt{E/\rho }$ of silicon is about two larger than other materials. However, from the realized large-aperture Si based micro scanner [5,10,11,12], mirror size of silicon based micro-scanner is below 1 cm and the frequency is less than 350 Hz. The metal based micro scanner is more robust than MEMS devices due to its ductile properties in comparison with brittle silicon substrates [15].

For most metal material, the ratio $\sqrt{E/\rho }$ is approximately equal. Therefore, micro-scanners with the same size but different metal material have approximately the same resonance frequency. From Equation (5), we can see that for the micro-scanner with the same size, the shear stress is proportional to shear modulus *G*. From Table 2, we can see that Young modulus of stainless-steel is about 1.7 times larger than Ti-alloy. Compared with the stainless-steel based micro scanner, the Ti-alloy based micro scanner has lower shear stress and higher tensile strength. The maximum scanning angle of the Ti-alloy based micro scanner is significantly larger than the stainless-steel based micro mirror.

Relative shear strength can be defined here which can be given as:

(16) $${\left[\tau \right]}_{r}=\frac{\left[\tau \right]}{G}$$

From Equation (15), we can calculate the relationship between maximum scanning and shear strength:

(17) $$\theta_{max}\leq k_{size,2}{\left[\tau \right]}_{r}$$

where *k_size,_*_2_ is a coefficient which is only related to size.

For most material, tensile strength can be obtained from research and we calculate the relative tensile strength [σ]/E instead. Table 1 demonstrates that Ti-alloy has the maximum relative strength. Ti-alloy substrate is chosen to design the micro scanning mirror.

Our device has the advantages of mirror plate size and scanning frequency. In comparison to the Galvo Scanner (see Table 3) which is usually occupied in the LiDAR system, the Ti-alloy-based micro-mirror has comparable large apertures while the operating frequency is about an order of magnitude larger. Compared with large-aperture MEMS scanning mirror, the aperture, operating frequency and scanning angle are much larger. The MEMS mirror has the advantages of small size and low power consumption while it does not meet traditional LiDAR scanner’s demand like the large aperture. Our device is designed using the MEMS design method and it can link the gap between the traditional scanner and the MEMS scanning mirror. The Ti-alloy based micro mirror can meet the demand of the LiDAR system while it retains some advantages of the MEMS mirror like small size and low power consumption.

## 7. Conclusions

A novel, one-axis Ti alloy-based electromagnetic micro scanning mirror with a very large-aperture and rapid resonant frequency is presented in this paper. The micro mirror is designed for the demand of a MEMS based LiDAR system. Ti-alloy substrate is used to achieve larger aperture and faster scanning frequency and a pair of moon-like magnets are used to achieve larger optical scanning angle with low actuation current. The Ti alloy-based electromagnetic micro-scanner has very large-aperture (12 mm) and rapid scanning frequency (1.24 kHz). The optical scanning angle can reach 26 degrees when the actuation current is 250 mApp. In comparison with the Galvo Scanner which is usually occupied in the LiDAR system, the Ti-alloy-based micro-mirror has comparable large apertures while the operating frequency is about an order of magnitude larger. The size of the micro-scanner reached 12 mm which is much larger than similar MEMS scanning mirrors. The Ti alloy-based large-aperture micro scanner will speed up the micro mirror’s application in LiDAR.

## Figures and Tables

**Table 1:** Parameters of the Ti alloy based micro scanner.

Parameter	Symbol	Value	Parameter	Symbol	Value
Radius of mirror substrate	*R_s_*	6 mm	Turns of coil	*N*	200
Thickness of mirror substrate	*t_s_*	0.4 mm	Width of torsional beam	*w*	1 mm
Radius of mirror plate	*R_m_*	6 mm	Depth of torsional beam	*h*	0.4 mm
Thickness of mirror plate	*t_m_*	0.2 mm	Length of torsional beam	*l*	5.8 mm
Internal radius of coil	*R_c_*_1_	6 mm	Internal radius of magnet	*R_mag_*_1_	7 mm
External radius of coil	*R_c_*_2_	5 mm	External radius of magnet	*R_mag_*_2_	12.5 mm
Thickness of coil	*t_c_*	0.5 mm	Thickness of magnet	*t_mag_*	7 mm

**Table 2:** Parameters of some common materials [21].

Material	Young Modulus (GPa)	Density (kg·m^−3^)	Poison Ratio	$\sqrt{E/\rho }$ (m/s)	Tensile Strength (MPa)	[*σ*]/*E* (1 × 10^−3^)
Silicon	150–170	2.33 × 10^3^	0.28	8023–8541	1320 ^1^	8.3–8.8
SUS304 Stainless steel	193	7.9 × 10^3^	~0.3	4943	520	2.7
TC4 Ti-alloy	116	4.5 × 10^3^	0.34	5077	895	7.7
7050 Al alloy	68.5	2.7 × 10^3^	~0.3	5036	485	7.1

^1^ The size of the Silicon specimen is 3000 × 250 × 24.5 μm^3^ [14].

**Table 3:** Features of our device in comparison with Galvo Scanner and large-aperture MEMS scanning mirror.

Type	Diameter/mm	Frequency/Hz	FOV/Deg	Driving Voltage/Current	Angle Measurement
Our device	12	1240	26	250 mA	PSD
Fraunhofer [5]	2.5 × 9.5	250	30	180 Vpp	Photodiodes
Huikai Xie [10]	10 × 10	234	10	7 Vpp	NO
Mirrorcle tech. [11,12]	5	330/334	10 × 10	157 V	NO
Galvo Scanner [22]	10	150	40	1.25 A rms	YES
